# Poly (ADP-ribose) polymerases as PET imaging targets for central nervous system diseases

**DOI:** 10.3389/fmed.2022.1062432

**Published:** 2022-11-10

**Authors:** Jie Tong, Baosheng Chen, Peng Wen Tan, Stephen Kurpiewski, Zhengxin Cai

**Affiliations:** Yale PET Center, Department of Radiology and Biomedical Imaging, Yale School of Medicine, New Haven, CT, United States

**Keywords:** positron emission tomography, PARP1, radiotracers, BBB, neurodegenerative disease

## Abstract

Poly (ADP-ribose) polymerases (PARPs) constitute of 17 members that are associated with divergent cellular processes and play a crucial role in DNA repair, chromatin organization, genome integrity, apoptosis, and inflammation. Multiple lines of evidence have shown that activated PARP1 is associated with intense DNA damage and irritating inflammatory responses, which are in turn related to etiologies of various neurological disorders. PARP1/2 as plausible therapeutic targets have attracted considerable interests, and multitudes of PARP1/2 inhibitors have emerged for treating cancer, metabolic, inflammatory, and neurological disorders. Furthermore, PARP1/2 as imaging targets have been shown to detect, delineate, and predict therapeutic responses in many diseases by locating and quantifying the expression levels of PARP1/2. PARP1/2-directed noninvasive positron emission tomography (PET) has potential in diagnosing and prognosing neurological diseases. However, quantitative PARP PET imaging in the central nervous system (CNS) has evaded us due to the challenges of developing blood-brain barrier (BBB) penetrable PARP radioligands. Here, we review PARP1/2's relevance in CNS diseases, summarize the recent progress on PARP PET and discuss the possibilities of developing novel PARP radiotracers for CNS diseases.

## Introduction

Poly (ADP-ribose) polymerases (PARPs) are a family of enzymes that catalyze the transfer of ADP-ribose to target macromolecules. PARPs play a pivotal role in a variety of cellular processes such as DNA damage repair, chromatin reorganization, genomic integrity, apoptosis, and inflammation (1). To date, 17 PARP family members encoded by different genes have been identified and found to have homology in the conserved catalytic domain (2). Among them, PARP1 is the dominant subtype of PARPs and performs greater than 90% of PARPs functions; while the roles of PARP2 and other PARP subtypes in DNA repair have not been well studied (3).

Since PARP1 is overactivated in many cancers, such as ovarian, breast, and oral cancers, impairing the DNA damage repair (DDR) pathway of cancer cells by inhibiting PARP1 activity turned out to be a promising cancer treatment strategy (4), as evidenced in the clinical settings where PARP1 inhibitors are administered to patients who have breast or ovarian cancer with BRAC1/2 mutation (HR deficiency) (5). Moreover, because the elevated PARP1 expression in response to oxidative/nitrosation stress has been found in many different neurological diseases, repurposing or developing novel PARP1 inhibitors as neurotherapeutic agents have piqued the interest of researchers (6).

**Figure 1:**
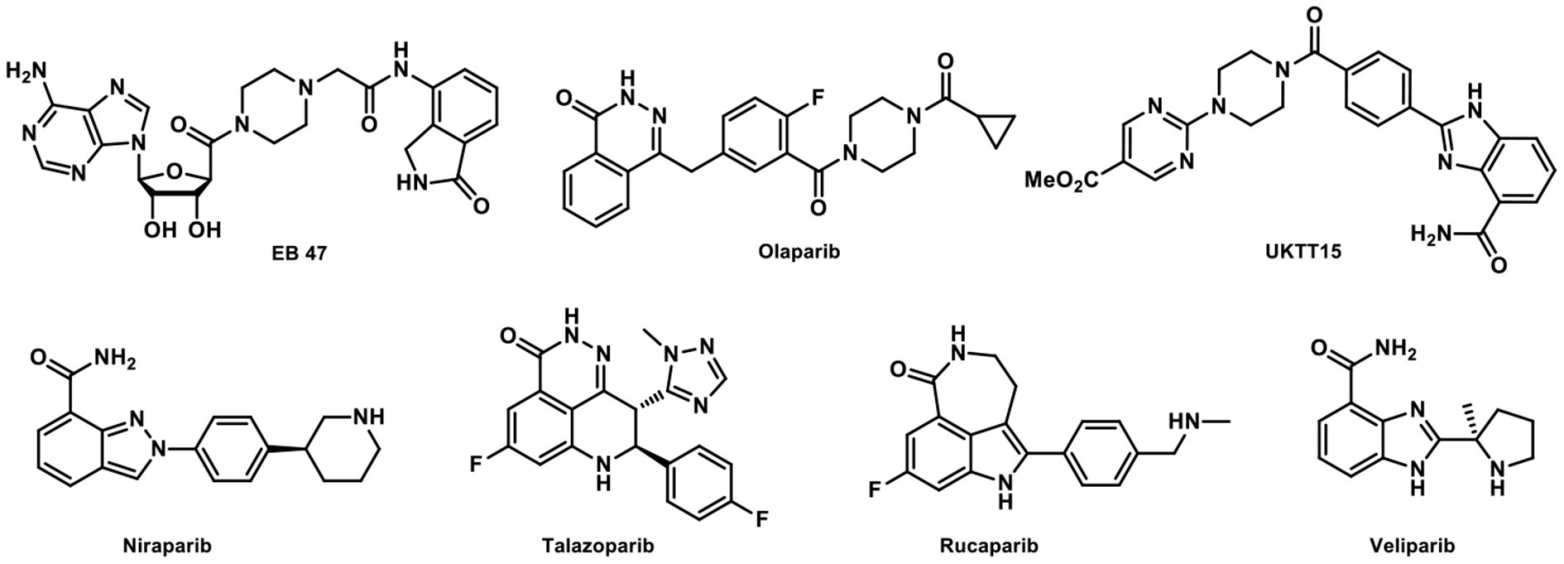
Representative PARP inhibitors.

In the past decade, four PARP inhibitors (PARPis), i.e., Olaparib, Rucaparib, Niraparib, and Talazoparib have been approved by US FDA for cancer treatment. Veliparib has been investigated in phase III trial with promising results (Figure 1) (7). There is also a growing interest in imaging PARP with fluorescent or radiolabeled PARPis to visualize and quantify PARP expression, as well as the pharmacokinetics and target engagement of PARPis. After successful clinical translation, PARP imaging is expected to improve patient stratification and allow us to monitor treatment responses (8). Radiolabeled PARP-targeted radionuclide therapeutic agents have also been proposed for the treatment of cancers with PARP overexpression, e.g., [^125^I]KX1 in ovarian cancer (9). In addition, the development of PARP specific PET imaging probes that could penetrate the blood-brain barrier (BBB) for the quantification of PARP in the central nervous system (CNS) would be groundbreaking as this would enable the exploration of the PARP-related pathological processes in various neurological diseases and the development of new therapeutic strategies (10).

PARP1 activation is one of the mediators of neuronal death under excitatory toxicity, oxidative stress, and ischemia. This type of neuronal death is termed “parthanatos” and can be ameliorated by PARP1 gene deletion or pharmacological inhibition. In several neurodegenerative diseases, i.e., AD, PD, Huntington's disease, and ALS, PARP1's pathological roles have been established, and the use of PARP1 inhibitors as treatment has shown some promise. In this review, we surveyed PARP PET imaging probes developed in the past decades including the recently developed PARP PET imaging probes for CNS diseases, discussed the relation between PARP1/2 and various neurodegenerative diseases, and call for the development of new PARP PET tracers for CNS imaging.

## Current PARP PET imaging probes

As PARP is overexpressed in many cancers, inhibiting PARP activity that would disrupt the DDR pathway of cancer cells has become a promising cancer treatment strategy (11). From 2014 to 2018, four PARPis were approved by FDA for the treatment of BRCA-mutated advanced cancers, and more PARP inhibitors are being tested in clinical trials. Tagging a PARPi with a radionuclide for PET (^11^C, ^18^F) or SPECT (^123^I) imaging could be used to evaluate PARP expression levels noninvasively, impacting clinical diagnosis, prognosis, disease staging, treatment monitoring, and early detection of treatment resistance (12).

**Figure 2:**
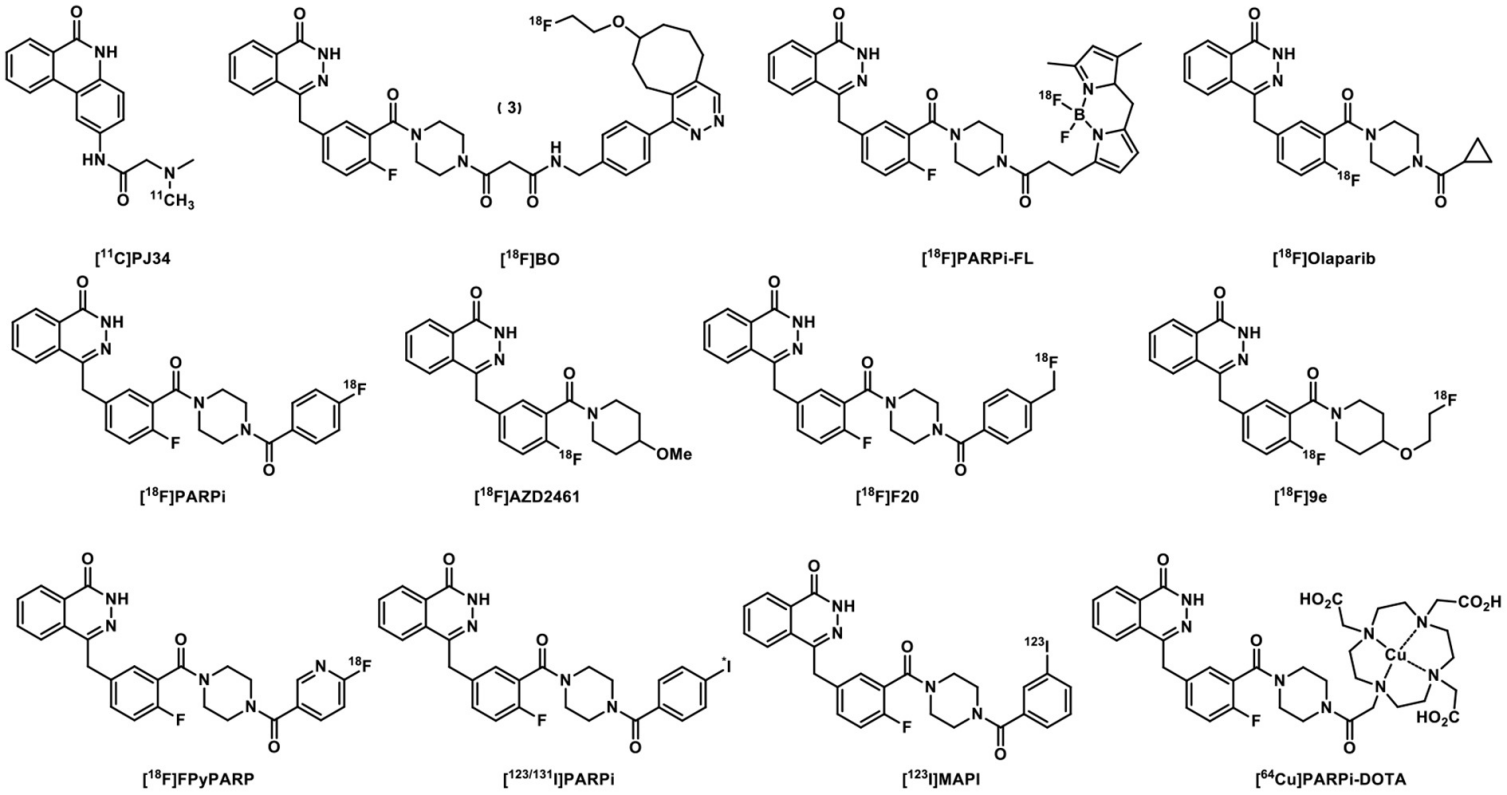
[^11^C]PJ34 and Olaparib-derived PARP-targeted radiotracers.

Indeed, over the past 17 years, we witnessed the development of numerous PARP-specific radiotracers, of which [^18^F]FTT and [^18^F]PARPi are the most advanced and have been translated to early clinical trials. About 17 years ago, [^11^C]PJ34 was the first to be tested for imaging the upregulated PARP1 expression in rodent models of diabetes (13). The radiolabeling of Olaparib, its analogs and derivatives has yielded a variety of radioligands with high binding specificity toward PARP (Figure 2). [^18^F]BO was developed based on the Olaparib scaffold to quantitate the therapeutic doses of Olaparib to inhibit PARP *in vivo* in ovarian cancer models and delineate the biodistribution of the drug (14). [^18^F]Olaparib was evaluated in PSN-1 xenograft-bearing mice and showed promise for imaging tumors and their responses to radiation (15). In comparison to [^18^F]FDG, the Olaparib analog [^18^F]PARPi is a promising PET tracer to image head and neck squamous cell carcinoma (16) and glioblastoma in mouse models with high tumor-to-background contrast (16, 17). [^18^F]PARPi-FL was shown to have high tumor uptake in U87 MG glioblastoma and the potential for optical imaging at the cellular resolution and systemic PARP PET imaging of malignant tumors (18). [^18^F]20 was assessed in mice bearing subcutaneously implanted glioblastoma xenograft as a PARP PET radiotracer and exhibited PARP specific binding allowing clear tumor visualization (19). [^18^F]AZD2461 targets PARP expression in a mouse model of pancreatic cancer *in vivo* and was evaluated in a variety of pancreatic ductal adenocarcinoma cell lines *in vitro* (20). Despite its high PARP1 binding affinity, [^18^F]9e, a derivative of AZD2461, was reported to be non-BBB penetrant in nonhuman primates (21). [^18^F]FPyPARP was designed and synthesized to improve the renal clearance profile of the PARP PET tracers used in humans, and it provided advantage for imaging abdominal lesions which might facilitate the development of new strategies in personalized cancer therapy (22). *Ex vivo* studies of [^123^I]PARPi, a potential PARP SPECT imaging agent, showed high specificity to PARP1 (23), while [^131^I]PARPi showed the potential as a PARP PET imaging agent for brain tumors (24). Another auger-emitting theranostic tracer, [^123^I]MAPi, has been studied in GBM models, showing high tumor uptake as well as survival benefit for treated animals (25). Besides these aforementioned non-metal labeled radiotracers, [^64^Cu]PARPiDOTA was evaluated in a mesothelioma mouse model (26).

**Figure 3:**
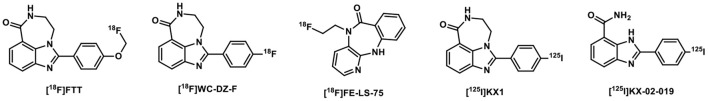
Rucaparib-based PARP-targeted radiotracers.

Rucaparib-inspired radiolabeled compounds also showed excellent PARP selectivity (Figure 3). [^18^F]FTT was used in patients with breast cancer, prostate cancer, and ovarian cancer and its SUV Max was associated with Gleason Grades and Decipher scores (27). [^18^F]WC-DZ-F was tested as a PET imaging agent for measuring PARP1 levels in prostate and other types of cancers (28). [^18^F]FE-LS-75 exhibited binding affinity of 0.2 μM and a logD within the suitable range for BBB penetration, and was selected as a candidate for molecular imaging of PARP1 using autoradiography and PET (29). [^125^I]KX1 showed high tumor uptake but low specific binding to PARP1 based on a blocking study using Olaparib and severe deiodination was also observed (30). Although [^125^I]KX-02-019 has specific binding in PARP1 KO cells and PARP2 KO cells, it has higher affinity for PARP2, and might be useful in studying PARP2-specific target occupancy by other PARPis (31).

**Figure 4:**
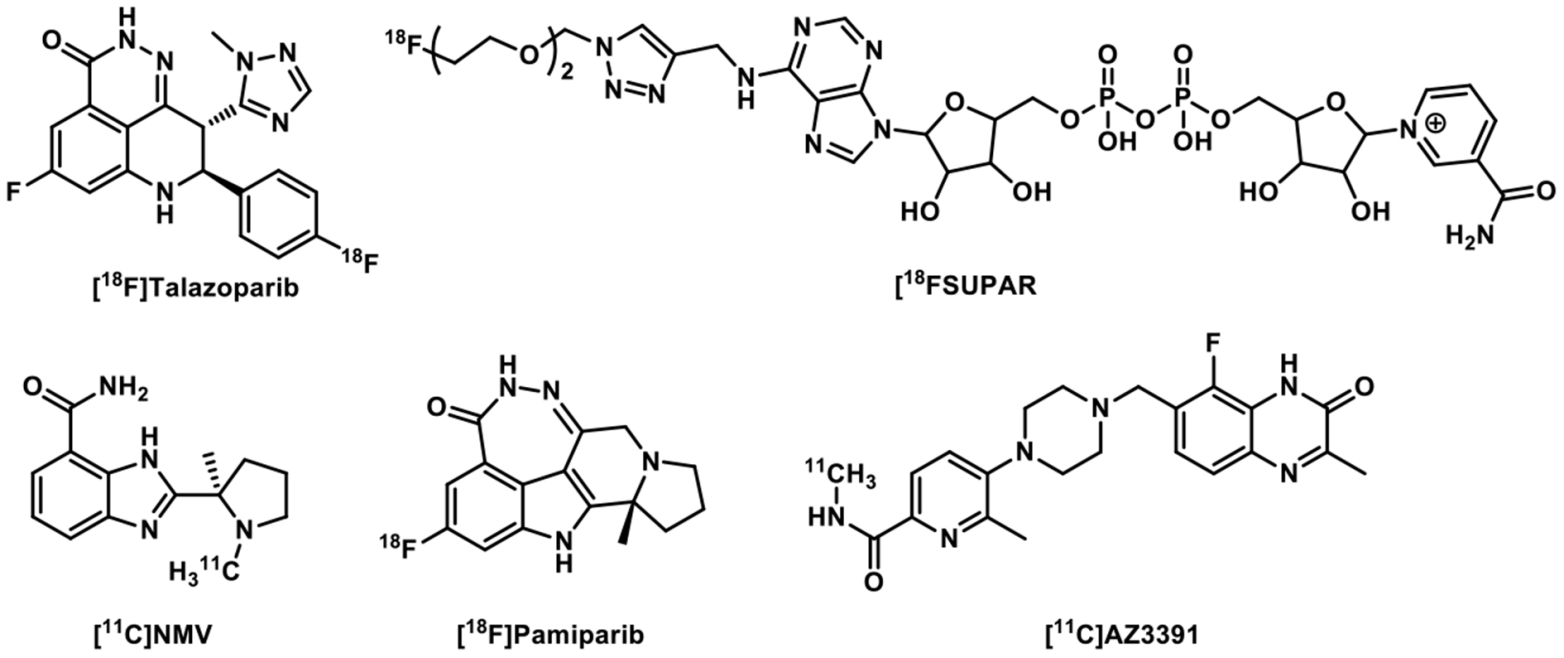
Other PARP-targeted radiotracers.

Moreover, many structurally novel PARP PET imaging probes have been reported recently, with some specifically aiming at brain PET imaging of PARP (Figure 4). [^18^F]Talazoparib was synthesized and evaluated in the murine PC-3 tumor model, showing high and persisted tumor uptake for up to 8 h post tracer administration (32). As a substrate of PARP1/2, [^18^F]SuPAR was developed to image PARP1/2 activity and its uptake was significant reduced in an orthotopic breast cancer model rather than in a subcutaneous model. Correspondingly, the [^18^F]SuPAR accumulation and PARP levels correlated well in tumor sections. However, its rapid pharmacokinetics and low serum stability (T_1/2_ < 1 h) astricted its use as a PARP1/2 PET tracer (33). While aforementioned tracers have low or no BBB permeability, [^11^C]NMV ([^11^C]PyBic) was first evaluated in the syngeneic RG2 rat glioblastoma model and nonhuman primates as a first BBB permeable PARP1/2 PET radioligand (34). [^18^F]Pamiparib was studied in rodents and nonhuman primates and was found to be a brain penetrable PARP1 tracer, albeit with lower brain uptake than [^11^C]PyBic (35). [^11^C]AZ3391 was identified as a promising BBB permeable PARP1 PET radioligand lead and has been preclinically validated through *in vitro* and *in vivo* imaging experiments (36).

As PARPs have been defined as therapeutic targets for cancers, metabolic, inflammatory, and neurological diseases, PARP PET tracers allow direct measurement of PARP expression in patients to enhance patient stratification, quantify target engagement by PARP inhibitors, and monitor treatments. So far, most PARP tracers were shown to have poor BBB penetration. Recent research developments are more inclined toward PARP tracers that have high brain uptake for diagnosing brain tumors. Furthermore, PARPs have received considerable attention as potential targets to treat metabolic, inflammatory, and neurological disorders. Correspondingly, the development of effective, sensitive, and definitive PARP tracers will advance our understanding of various CNS diseases via PARP PET imaging.

## PARP1 in CNS diseases

**Figure 5:**
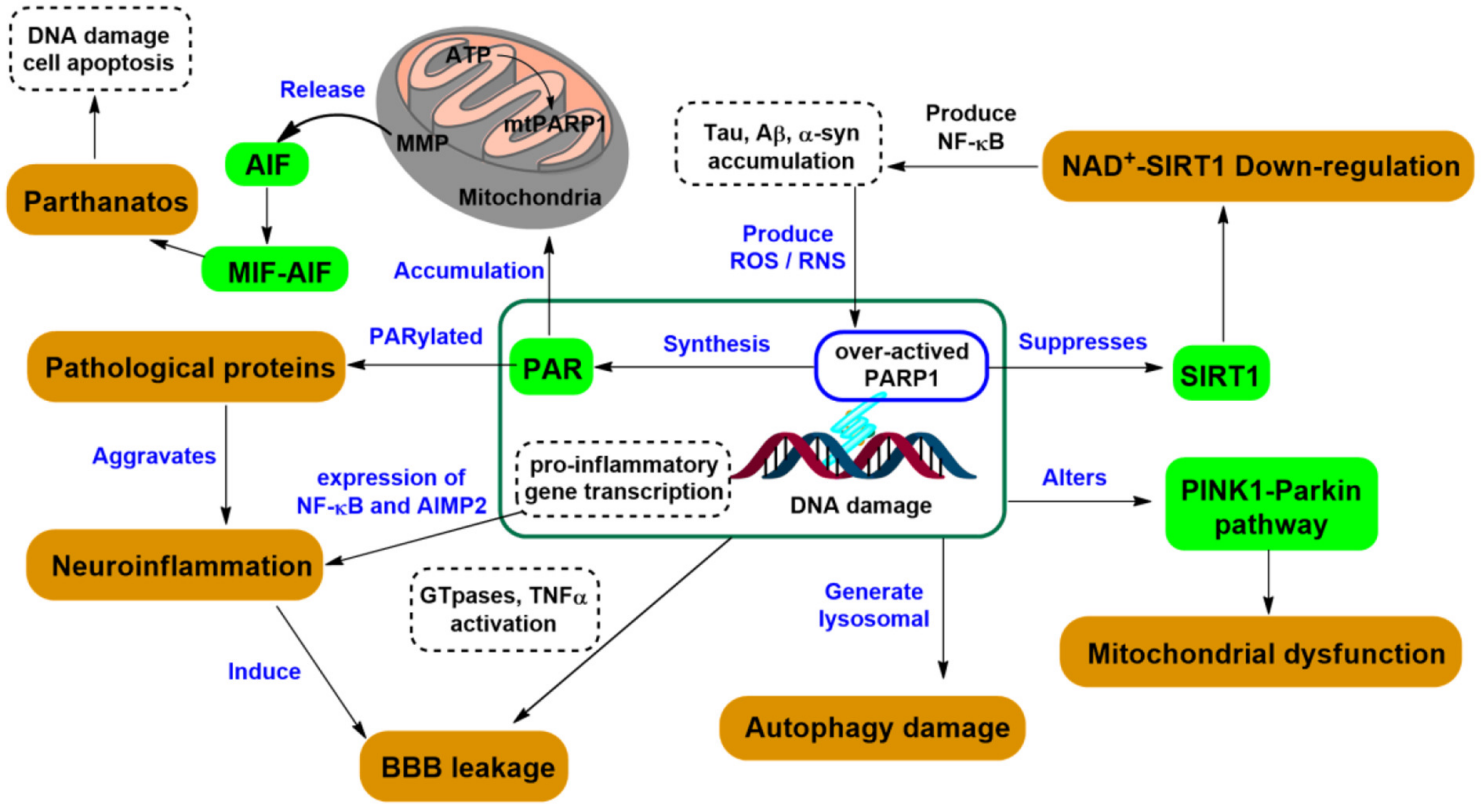
PARP1-related signaling pathways in neurodegeneration.

The causes of CNS diseases can be multifaceted with contributing factors from immunity, trauma, aging, congenital disabilities (37), as well as mutations in DNA repair factors (38), particularly those age-associated (39). PARP1 can affect the CNS differently depending on the cell type and the degree of DNA damage. DNA damage results in the activation of PARP1 and its participation in DNA repair (Figure 5). Nevertheless, this process consumes nicotinamide adenine dinucleotide (NAD^+^) to produce a branched polymer of ADP-ribose (PAR) on the targeted macromolecules (40). Consequently, PAR formation on histones and on enzymes can block sister chromatid exchange and aid base-excision repair. This affects the action of transcription factors, especially the role of nuclear factor κB, thereby promoting inflammation. Extensive PARP1 activation can stimulate neuronal death via NAD^+^ depletion and release of apoptosis-inducing factor (41). Thus, because PARP1 activation plays an important role in neuronal death during excitotoxicity, ischemia, and oxidative stress, exogenous pharmacological inhibition can significantly improve neuronal survival under these condition (42). Herein, we summarized the relationship between different types of CNS diseases and PARP1, focusing on the roles of PARP1 in neurodegenerative diseases.

PARP1 has a major regulatory role in vascular disorders but its regulatory mechanism remains unclear (43). Because (44) PARP1 dependent cell death plays a pivotal role in the progress of stroke, pharmacological inhibition of PARP1 can eliminate inflammation, protect neurons, regulate the translocation of apoptosis-inducing factor, and improve recovery of neurological functions in ischemic stroke (45, 46). Subarachnoid hemorrhage (SAH), an acute cerebrovascular disease is often associated with high mortality rate. Recent studies indicated that inhibiting PARP1/apoptosis-inducing factor signaling axis might facilitate the protective effect of electroacupuncture after subarachnoid hemorrhage (47). Hence, PARP inhibition by PJ34 could be a viable therapeutic revenue for SAH (48).

PARP1 has an impact on the expression of a range of inflammatory cytokines, such as IL-1β and TNF-α, and promote neuroinflammation, which could result in meningitis, encephalitis, polio, and epidural abscess. While the activation of PARP1 mediates meningitis-associated CNS complications, the disruption of the PARP1 gene or the inhibition of PARP1 could improve the clinical status of the infected mice (49). PARP1 activation has also been identified in experimental allergic and virus or bacterial induced encephalitis (41), with PARP inhibitors being identified to be anti-inflammatory (50).

Neurons are disconnected after traumatic injury, and their functional recovery is limited by poor axonal regeneration (51). Although the functions of PARP1 in the processes of myelination and remyelination remain elusive, PARP1 can limit axonal regrowth, indicating that inhibition of PARP may possess therapeutic values and boost neurological recovery after CNS trauma (52). PARP1-mediated PARylation is sufficient for oligodendrocyte progenitor cell differentiation (53), and PARP1 polymorphisms was regarded as one of the potential risk factors for spinal cord injury (SCI) in a clinical study (54). Besides, brain or spinal cord tumors remain difficult types of cancers to treat, such as glioblastoma (GBM), even after four PARP inhibitors were approved by FDA as PARP1 overexpression is present in various cancers (55). For other diseases that are resulted from structural damage of the nervous system, such as Bell's palsy, carpal tunnel syndrome, cervical spondylosis, Guillain-Barré syndrome, and peripheral neuropathy, PARP affects neuronal recovery albeit no direct evidence exists to prove the exact roles of PARP in these pathological changes.

Dysfunction is also a common pathological manifestation of the CNS diseases, such as epilepsy, dizziness, neuralgia, but its complex etiology remains perplexing. In epilepsy, seizures tend to recur with no immediate underlying causes (56), but it was shown that PARPis exert neuroprotective effects in epileptic rats through apoptosis-inducing factor and Akt signaling (57, 58). In advanced diabetic neuropathy, PARPis and PARP gene deficiency reduced intraepidermal nerve fiber loss and neuralgia (59), and attenuated chemotherapy-induced painful neuropathy (60). On the contrary, dizziness is a common side effect of PARP inhibitors, as reported at a frequency of 17% for niraparib, 15% for rucaparib, and 13% for olaparib (61). However, there is no denying that a better understanding of their effects on the nervous system will provide better guidance for improved treatment.

Neurodegenerative diseases are characterized by progressive memory loss and functional impairment, and are associated with neuroinflammation, autophagy dysregulation, and SIRT1 inactivation. PARP1 is linked to a group of stress signals that originate from inflammation and dysregulation of autophagy (40). Studies have revealed that Aβ enhances PARP1 activity in Alzheimer's disease (AD) patients' brain, with concurrence of reactive oxygen species (ROS) and active nitrogen species (RNS). This series of events are associated with the formation of Aβ plaques and tau tangles, thus aggravating AD symptoms (62). It is believed that PARP1 plays a key role in the inflammatory process associated with AD. Activated PARP1 enhances DNA binding with NF-κB in microglia and induces inflammatory responses in AD pathology, while inhibition of PARP1 by PJ34 slowed pathological progression in mouse models (63). Additionally, PARP1 competes for a common NAD^+^ pool with SIRT1 (Sirtuin 1), which is a neuroprotective factor in multiple AD models that inhibits Aβ accumulation and alleviates age-related cognitive decline (64). Moreover, inhibition of PARP1 enhances BBB integrity by promoting tight junction protein expression and reducing endothelial dysfunction caused by neuroinflammation, thus attenuates cognitive impairment (65). In retrospect, these findings posit PARP1 as a critical player in AD pathogenesis and progression.

In a rodent model of Parkinson's disease (PD), recombinant α-synuclein preformed fibrils (α-syn PFF) induced the activation of PARP1, which promoted the formation of more pathologic α-syn aggregates that eventually led to cortical neuronal death (66). Importantly, PARP1 inhibition increased autophagy activity and the degradation of pathological α-syn (67). Besides, activated PARP1 can regulate several pathological mechanisms in PD, including neuroinflammation, abnormal sleep rhythm, mitochondria dysfunction, and mitophagy dysregulation (68). Thus, PARP1 probably plays a crucial role in the PD pathogenesis, entangling with pathological α-syn.

Amyotrophic lateral sclerosis (ALS), is a motor neuron degenerative disease, with potential etiologies from disproportionate metal handling, oxidative damage, neurofilament abnormalities, and protein aggregation (69). Evidence suggested that PARP1 expression was increased in patients with sporadic ALS (70) and in the ALS G93A mouse model (71), which could be ameliorated using PARPis. These results underscore the potential of PARPis in ALS treatment.

The role of PARP1 overactivation in the pathogenesis of Multiple sclerosis (MS) is unclear. However, significant PARP1 activity was found in plaque regions of the brain of marmoset monkeys with experimental autoimmune encephalomyelitis (EAE), and PARP1 activity was significantly increased in the astrocytes, microglia, endothelial cells, oligodendrocytes, and neurons surrounding the plaque (72). In addition, the PARP1 inhibitor PJ34 attenuated DCS migration, demyelination, and nerve damage in C57Bl chronic EAE mice and SJL chronic EAE recurrent mice (73). Henceforth, PARP1 likely plays a vital role in the progression of MS, and PARPis might be potential drug candidates for treating MS.

Patients diagnosed with Huntington's disease (HD) exhibited enhanced expression of PARP in their brains, indicating the involvement of PARP in HD associated neuronal apoptosis (74). It has also been reported that in a transgenic mouse model of HD, PARP1 inhibitor INO-1001 can increase survival and reduce the degree of abnormal neurobehavior in mice (75). Similarly, different types of spinocerebellar ataxias (SCAs) are closely associated with DNA repair and neuronal metabolism (76), and SCA7 patients displayed increased PARP1 in cerebellar neurons (77). These findings suggest the potential use of PARPis in the treatment of HD and SCA.

Currently, there has been no study that demonstrated the association of PARP with other neurodegenerative diseases, such as Pick disease. Generally, neurodegenerative diseases are characterized by cognitive impairment or motor disability, but most are associated with aging. Oxidative stress, DNA damage, and metabolic abnormalities are contributing factors for the occurrence and exacerbation of these diseases. *In vivo* or *in vitro* experiments have demonstrated the expression and activity of PARP1 in pathological sites is related to the development of neurodegenerative diseases. PARP1 can induce parthanatos, prevent autophagy, accumulate toxic proteins, and play a role in DNA repair, cellular energy metabolism, and neuroinflammation. Given the complex causes of these diseases and the lack of specific treatment options, it is necessary to understand the specific molecular pathway underlying the disease mechanisms to develop new therapeutic approaches to treat multiple neurogenic diseases effectively. Although PARPis are only recently approved as drugs for the treatment of cancers, the outstanding neuroprotective potential of PARPis warrants further investigations and translation. The development of PARPis that can penetrate BBB will not only pave the way for developing new therapeutic strategies to treat CNS disorders but also serve as a beacon for early disease detection through PET/SPECT imaging.

## Perspectives on PARP1 PET in CNS imaging

PET imaging of kinases associated with CNS diseases can address some controversial issues, such as the occurring sequences of pathophysiological biomarkers, energy and neurotransmitter system malfunctions, and their relative impact on pathogenesis and disease progression (78). In AD, the most common neurodegenerative disease, hippocampal atrophy, temporoparietal FDG hypometabolism, and increased amyloid plaque and tau tangle deposition have been suggested as diagnostic criteria for amnestic forms of AD (79). It has been proven that PET imaging played a crucial role in studying the underlying pathophysiological hypotheses regarding AD and has largely contributed to the evolution of diagnostic methods (80). PET imaging of PARP1 could reveal the roles of PARP1 in the pathophysiological alterations in AD brains, such as excessive aggregation of misfolded Aβ, hyperphosphorylated tau proteins, neuroinflammation activation, cholinergic deficit, impaired glucose utilization and synaptic dysfunction, and facilitate the translation of PARP1 as a therapeutic target in AD.

Compared with AD PET imaging, the arsenal for PD PET imaging has been relatively scarce. Although PET imaging of the dopaminergic system in striatum has been widely used, studies have shown that dopamine transporters (DAT) imaging alone does not sufficiently reflect nigral neurodegeneration in some PD patients (81). Therefore, to justify the use of dopaminergic medications, several radiotracers such as Fluorodopa (F-Dopa) have been developed to determine striatal dopamine terminal dysfunctions (82). PET being at its infancy at detecting other types of CNS diseases, it is critical to develop PET imaging probes for disease relevant targets.

As PARP1 activation is a crucial mediator of neuronal death under excitatory toxicity, oxidative stress, ischemia, it is essential to understand the dynamic change of PARP1 in these neurological diseases and elucidate the pathogenic mechanisms (83). PET imaging of PARP1 will significantly increase our understanding of the etiology and progression of neurodegenerative diseases and facilitate the discovery and development of novel CNS disease therapies. Therefore, it is of great significance and crucial to develop PARP1 PET probes with high specificity and selectivity, which can penetrate the BBB and have appropriate pharmacokinetics.

Future breakthroughs in the development of new PET tracer candidates will continue to advance our understanding of CNS diseases, so as to provide a sensitive and quantitative tool for early diagnosis, prognosis, and assessing therapeutic effects of therapeutic drug candidates.

## Author contributions

BC, ZC, and JT contributed to the conception and design of the review. JT wrote the first draft of the manuscript. JT, ZC, BC, SK, and PT revised the manuscript and approved the final version. All authors contributed to manuscript revision, read, and approved the submitted version.

## Funding

ZC was supported by grants from the National Institutes of Health (NIH) R03CA249569, R01AG058773, R01NS123183, R01AG069921, R21EB027872, and R21CA252587.

## Conflict of interest

The authors declare that the research was conducted in the absence of any commercial or financial relationships that could be construed as a potential conflict of interest.

## Publisher's note

All claims expressed in this article are solely those of the authors and do not necessarily represent those of their affiliated organizations, or those of the publisher, the editors and the reviewers. Any product that may be evaluated in this article, or claim that may be made by its manufacturer, is not guaranteed or endorsed by the publisher.

## Author disclaimer

The content is solely the responsibility of the authors and does not necessarily represent the official views of the National Institutes of Health.
